# Portable XRF Technology to Quantify Pb in Bone *In Vivo*

**DOI:** 10.1155/2014/398032

**Published:** 2014-11-27

**Authors:** Aaron James Specht, Marc Weisskopf, Linda Huiling Nie

**Affiliations:** ^1^School of Health Sciences, Purdue University, West Lafayette, IN 47907, USA; ^2^Harvard School of Public Health, Boston, MA 02115, USA

## Abstract

Lead is a ubiquitous toxicant. Bone lead has been established as an important biomarker for cumulative lead exposures and has been correlated with adverse health effects on many systems in the body. K-shell X-ray fluorescence (KXRF) is the standard method for measuring bone lead, but this approach has many difficulties that have limited the widespread use of this exposure assessment method. With recent advancements in X-ray fluorescence (XRF) technology, we have developed a portable system that can quantify lead in bone *in vivo* within 3 minutes. Our study investigated improvements to the system, four calibration methods, and system validation for *in vivo* measurements. Our main results show that the detection limit of the system is 2.9 ppm with 2 mm soft tissue thickness, the best calibration method for *in vivo* measurement is background subtraction, and there is strong correlation between KXRF and portable LXRF bone lead results. Our results indicate that the technology is ready to be used in large human population studies to investigate adverse health effects of lead exposure. The portability of the system and fast measurement time should allow for this technology to greatly advance the research on lead exposure and public/environmental health.

## 1. Introduction

Lead (Pb) exposures have decreased with the removal of Pb from gasoline. However, Pb exposure and toxicity remains an important public health issue. Certain populations in the USA as well as in many developing countries still experience high exposures. Moderate to high levels of exposure remain commonplace globally. Recent research also shows significant health effects at low exposure levels. In children, an inverse association between blood Pb level and cognitive abilities is observed at very low blood Pb concentrations, and the Pb associated intellectual decrement was steeper at low blood Pb levels than at higher blood Pb levels [1–3]. In adults, it has been shown that even low Pb exposures are associated with significant health effects among nonoccupationally exposed populations [4–9]. Traditionally, blood Pb is used as a biomarker to determine Pb exposures, but blood Pb has a half-life of 30 days and therefore correlates less well with long-term exposure than does bone Pb, for which the half-life is several years to decades [10, 11]. Cd-109 induced K X-ray fluorescence (KXRF) technology has been used to measure Pb in bone for over two decades and has made significant contributions to the study of associations between long-term cumulative Pb exposure and adverse health outcomes [4, 5, 7, 12–14]. However, the system requirement of a radioactive source, long acquisition times, and a sizeable space for the equipment limits this research to very few groups who possess this technology. In a previous study, we demonstrated the validity of a portable XRF system that made use of Pb L X-rays to quantify Pb in bone [15]. Improvements to this portable system's geometry and detector have been made, which decrease the minimum detection limit and make the device more compatible for use* in vivo*. The new system was tested with phantoms to determine the minimum detection limit of the device. Tests with phantom, goat bone, and cadaver bone samples were used to determine the accuracy of the device in determining bone Pb concentrations. Pb L X-rays, which have relatively low energies, have greater soft tissue attenuation for the signals and hence the correction for this is a significant issue. To this end, new calibration methods are being explored in this study to establish a more accurate approach to quantify the Pb in bone* in vivo*.

## 2. Materials and Methods

### 2.1. KXRF Bone Pb Measurement System

KXRF technology is used in this study to validate the results found with the portable XRF device. The setup of the device is the same as that used in previous studies [16, 17]. The system uses four 16 mm diameter high-purified germanium (HpGe) detectors with 10 mm thickness, four feedback resistance preamplifiers, four digital signal processing systems, and a computer. A 135 mCi ^109^Cd source is used to irradiate tibia bone or bone equivalent samples to produce the Pb K X-rays. The bone Pb measurements were taken for 30 minutes with the HpGe detector and then processed with digital electronics. The spectra were analyzed using an in-house peak fitting program and the final Pb concentrations were calculated [17–19]. The whole body effective dose from this system was measured to be 0.26 *μ*Sv for adults [20].

### 2.2. Portable XRF Device

Two customized portable XRF devices were used in this project (XL3t and XL3t GOLDD+, Thermo Fisher Scientific Inc., Billerica, MA). The XL3t device used in our previous study [15] is used in this study for a comparison to determine how the improvements in the device technology impact the measurements. Both devices have an energy span up to 50 keV. Previously the device was equipped with a thermoelectric cooled Si PIN diode with 8 mm^2^ area and 1 mm thickness. The device also has a tube voltage of up to 50 kV, a current of up to 40 *μ*A, and various filter combinations. The new device (XL3t-GOLDD+) has a more compact and optimized geometry. It uses a thermoelectric cooled silicon drift detector with a 25 mm^2^ area and 1 mm thickness. The devices were customized so that the voltage of the X-ray tube, the current of the tube, and the filter combinations could be selected to allow the best performance for* in vivo* measurement of Pb in bone. In our experiment, we used a measurement time of 3 minutes. Based on our previous study, by adjusting values for increased measurement time and tube current, we estimated the entrance skin dose of the system was 31 mSv to a 1 cm^2^ area and the whole body effective dose was 3.6 *μ*Sv [15]. This can be compared to the whole body effective dose for a standard AP chest X-ray of about 100 *μ*Sv.

### 2.3. Soft Tissue and Bone Equivalent Phantoms

Soft tissue and bone equivalent phantoms were used in this study to determine the sensitivity of the device and to calibrate the system. Lucite plate phantoms were used to simulate soft tissue over bone by placing the Lucite over the flat surface of the bone phantoms in increments of 1 mm up to 5 mm of Lucite thickness. Cylindrical Pb doped phantoms made of plaster-of-Paris were used to simulate bone with Pb concentrations ranging from 0 to 100 ppm (0, 5, 10, 15, 20, 30, 50, 75, and 100 ppm). These measurements were made from the flat base of the phantom. In our new calibration method (i.e., background subtraction), the Compton scattering peak was used to determine the background under the Pb L X-ray peak and the attenuation of the Pb signal. Hence, MC simulations were performed to test the differences between plaster-of-Paris and bone and Lucite and soft tissue in terms of Pb over Compton signal. No significant differences in XRF spectra were found between plaster-of-Paris with Lucite and bone with soft tissue. Thus, the phantom measurements were used to accurately calibrate the system, correlate the Compton peak counts with soft tissue thickness, and calculate the detection limit of the system.

### 2.4. Goat Bone and Cadaver Bone Measurements

Four goat bone samples and ten human cadaver tibia bone samples were measured with the device as well. The samples had varying Pb concentrations that were measured using KXRF. The bones were all vacuum-sealed in plastic bags and labeled for ease of measurements. For goat bone, measurements were made at 0, 1, 2, 3, 4, and 5 mm of Lucite and for bare cadaver bone, measurements were made using 0, 1, 2, and 3 mm of Lucite for comparison between portable XRF devices and KXRF. The cadaver bones measurements did not include 4 and 5 mm of Lucite due to the difficulty of adjusting the geometry in these situations. Three cadaver bone samples had intact soft tissue over them and were measured through the soft tissue. The cadaver and goat bone samples were taken to give a more realistic sense of the device capabilities for* in vivo* use by attempting to replicate the difficulties from increased attenuation with LXRF energies and soft tissue thickness.

### 2.5. Spectrum Analysis

#### 2.5.1. Background Subtraction

The spectrum was analyzed using a background subtraction method described in detail in our previous study [15]. In summary, the method is focused on deriving two functions that will enable us to estimate the Pb concentration. First, we define the relation of the background in the Pb peak areas to Compton peak area counts for 0 to 5 mm of Lucite. Second, we define the relation between Pb L-X ray signal and Compton peak counts for 0 to 5 mm of Lucite.

The background at 0 ppm will relate to scatter events, which will be the main contribution to background in the spectrum. The Compton scattering peak will give a correlation to the amount of scatter events in the spectrum and the background throughout the spectrum. Thus, defining a function that will relate the Compton peak counts and the 0 ppm background can be feasibly used to determine the background under the Pb L-X ray peaks.

The net signal will decrease with increasing Lucite thickness because of an increase in attenuation of the signal as well as distance from the bone. The Compton peak has been shown to accurately correlate with Lucite thickness through the increase in scatter events created by additional Lucite. Since the attenuation and distance will increase directly with Lucite thickness, we can correct each Pb peak by relating it with Compton peak counts. This function can then be used to accurately determine the signal attenuation that occurs in each spectrum. Then, one can determine from the spectrum the net counts coming from Pb in the sample and relate that to a known signal concentration value to compute the final sample concentration. In our study, as a modification to this method for better applicability for use* in vivo*, we tried two modifications in addition to the original method.

#### 2.5.2. Bone Calibration

Our second method is similar to the background subtraction method. Instead of making an adjustment to match the phantom calibration, we made a calibration with goat bones of known validated Pb values. For this calibration we used four goat bones with concentrations of 1, 13, 16, and 31 ppm of Pb at varying Lucite thicknesses as our calibration standards for the background subtraction method. For our 0 ppm data, we extrapolated from these values for each Lucite thickness from the 1 ppm bone and used our highest concentration bone at 31 ppm to replace the 100 ppm phantom in the background subtraction method.

#### 2.5.3. Bone Adjustment

**Figure 1:**
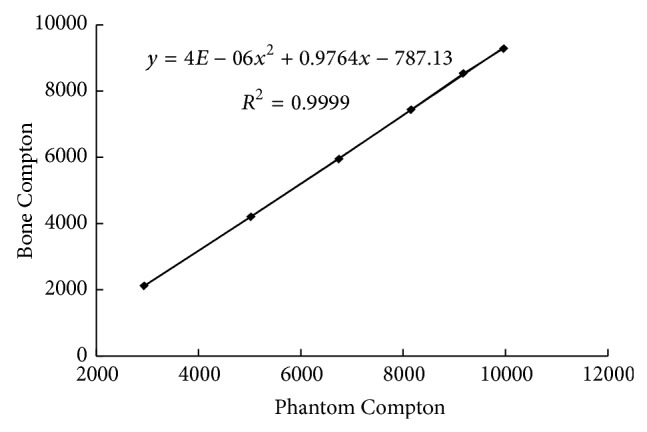
Compton scattering peak counts from bone versus that from phantom with Lucite thicknesses increasing by 1 mm with each point.

For this calibration method, we found the difference in Compton peak between actual bone samples and our calibration phantoms for varying Lucite thicknesses. These peaks change both with bone versus phantom and with varying Lucite thicknesses because of the densities and effective *Z* values of the materials. This comparison can be seen in Figure 1. By making this comparison we were able to apply a fit to this change and using this fit, we can apply the change between phantom and bone to any bone data we take, thus correcting it for use with phantoms. After this correction, we can apply the background subtraction method used with phantoms.

#### 2.5.4. Traditional Peak Fitting

We implemented a traditional peak fitting method primarily for comparisons between our novel calibration methods and the calibration methods used in previous studies of LXRF bone Pb measurement systems [21]. The peak fitting was carried out using MatLab. The peaks were fitted with a Gaussian peak with an exponential background. The fitting was performed on the Pb *L*_*α*_ and *L*_*β*_ peaks for phantoms at different Lucite thicknesses to determine the signal associated with each concentration. Then the same function was used to fit the *L*_*α*_ and *L*_*β*_ peaks associated with our cadaver bone and goat bone samples and the corresponding concentration was determined based on the net counts in those peaks corrected for Lucite or soft tissue attenuation. In previous studies of LXRF technology, it was concluded that the technology was not suitable for* in vivo* bone Pb measurement due to the significant soft tissue attenuation [21]. We included traditional peak fitting results from our goat bone data to show the comparison to our novel calibration methods.

## 3. Results

### 3.1. Portable XRF Spectrum

**Figure 2:**
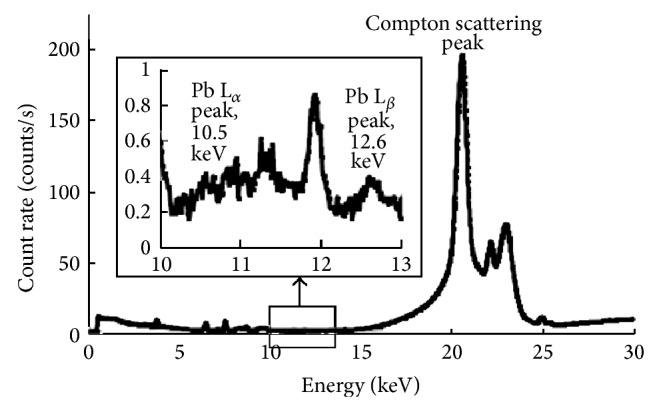
LXRF spectrum from cadaver bone with 1.3 mm soft tissue.

Figure 2 shows the resultant portable XRF spectrum from a measurement of an intact human cadaver bone with 1.3 mm soft tissue. As shown in the spectrum, the Compton scattering peak comes from the X-ray tube silver characteristic X-rays undergoing Compton scattering in our sample. This peak is a significant spectral feature and can be related to the background scattering events throughout the X-ray spectrum as we will demonstrate with our background subtraction calibration method in later results. This spectrum also demonstrates the difficulty in using traditional peak fitting methods, since with more soft tissue, there will be more background and the peaks will become increasingly noisier.

### 3.2. Detection Limit Comparison for New Portable XRF Devices

The measurements of the Lucite covered Pb doped phantoms were used to calculate the detection limit. The detection limit was calculated as

(1) $$\begin{array}{c} \text{DL}=2\times \sigma_{0\;\text{ppm}}=2\times \sqrt{\frac{1}{1/\sigma_{\alpha ,0\;\text{ppm}}^{2}+1/\sigma_{\beta ,0\;\text{ppm}}^{2}}}, \end{array}$$

where

(2) $$\begin{array}{c} \sigma_{\left(\alpha ,\beta \right)0\;\text{ppm}}=100\;\text{ppm}\times \frac{\sqrt{{\text{BKG}}_{0\;\text{ppm}}/180\;\text{s}}}{{\text{Gross}}_{100\;\text{ppm}}-{\text{BKG}}_{0\;\text{ppm}}}, \end{array}$$

where BKG_0 ppm_ is the background count rate under the *L*_*α*_ or *L*_*β*_ peak for the 0 ppm phantom and Gross_100 ppm_ is the total count rate under the *L*_*α*_ or *L*_*β*_ peak for the 100 ppm phantom.  Table 1 lists the detection limit of the portable XL3t GOLDD+ system and the older portable XL3t system. This comparison was taken at the same X-ray tube settings and filter on each device in order to demonstrate the improvements of the new system. This data was taken for 3 minutes at the same settings used for other measurements.

### 3.3. Correlation of Bone Pb Concentrations between KXRF and Portable XRF

Measurements were made to validate the portable XRF system against the standard KXRF systems for* in vivo* bone Pb measurements. Phantoms, goat bones, and cadaver bones with 0, 1, 2, and 3 mm of Lucite were measured by both systems, with goat bone also being measured at 4 and 5 mm of Lucite thickness.  Table 2 shows the measured phantom Pb concentrations at different Lucite thicknesses. The correlation (*R*-squared) between the expected concentrations and those measured with portable XRF system ranges from 0.991 to 0.999 for soft tissue thicknesses of 0 to 3 mm, demonstrating a good agreement of Pb concentrations determined by KXRF and portable XRF for bare and Lucite covered phantoms.

Tables 3 and 4 demonstrate the ability of the three calibration methods to quantify bare cadaver bone Pb values.  Table 3 shows the Pb concentration in bare cadaver bone calculated using the three calibration methods.  Table 4 shows the bone Pb concentrations for cadaver bone covered with 3 mm of Lucite. Without Lucite the calibration methods tend to be fairly similar, but with the introduction of more Lucite the bone adjustment method tends to get further from KXRF values by overestimating background levels. Bone calibration has a similar correlation, but with only 4 points on the calibration line and the highest point at 30 ppm the actual values tend to deviate from KXRF especially for higher Pb concentrations. Higher concentration standards are necessary to get visible signal while defining our function to correct for the inverse square and attenuation signal degradation as soft tissue increases. Background subtraction was the most reliable calibration method for higher Lucite thicknesses and lower Pb concentrations, which was determined using the correlation values for the cadaver bone evaluated at different Lucite thicknesses.

**Figure 3:**
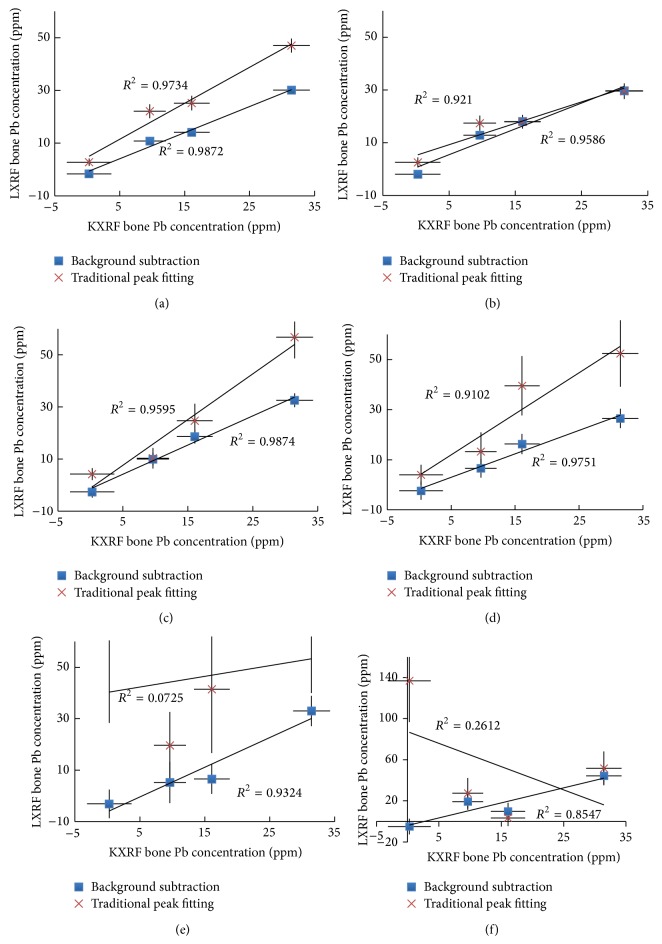
(a) KXRF versus LXRF for bare goat bone. (b) KXRF versus LXRF with 1 mm Lucite thickness over the goat bone. (c) KXRF versus LXRF with 2 mm Lucite thickness over the goat bone. (d) KXRF versus LXRF with 3 mm Lucite thickness over the goat bone. (e) KXRF versus LXRF with 4 mm Lucite thickness over the goat bone. (f) KXRF versus LXRF with 5 mm Lucite thickness over the goat bone.

Figures 3(a)–3(f) show the comparison of the correlations between goat bone Pb concentrations calculated by KXRF and portable XRF at Lucite thicknesses of 0–5 mm, with the Pb concentrations for portable XRF being calculated using traditional peak fitting or background subtraction. From the correlations, one can see that traditional peak fitting does fairly well for bare bone or at lower Lucite thicknesses, but with higher Lucite thicknesses the correlation falls off quickly due to the high background leading to the Pb peak being highly distorted especially at low concentrations. The chi-squared values for all the spectral fittings are close to 1, with the average chi-squared and standard deviation of the chi-squared value for these fits being 1.1 ± 0.4, which demonstrates that even for poor results the data is accurately represented by fitted function.

The data for cadaver bones with different Lucite thicknesses analyzed using the background subtraction method is presented in Table 5. The correlations (*R*-squared) between the concentrations obtained from KXRF and those from the portable XRF system range from 0.58 to 0.94 with Lucite thicknesses of 0 to 3 mm. The correlations between the Pb concentrations obtained from KXRF and portable XRF are worse for the cadaver bones than for the goat bones. This is mainly due to the lack of Pb concentration variation among the cadaver bones and geometry stability. Also, cadaver bone 6918 is an outlier (see Discussion).

To test the reliability and reproducibility of the technology for* in vivo* measurement, the cadaver bones with intact soft tissue were measured repeatedly. Only three such cadaver bones were available in our lab, so there are limited data for this test.  Table 6 shows the Pb concentrations from LXRF and KXRF for the three cadaver bones with intact soft tissue. The bones were measured nine times using the portable XRF device. The comparison in Table 6 demonstrates the abilities of the device in use through actual soft tissue. Using the Compton peak to determine soft tissue thickness, which was shown to be comparable to an ultrasound measurement in our previous paper [15], we found the intact soft tissue thicknesses for our three cadaver bone samples to be 1.3 mm for cadaver bone 7042, 4.1 mm for cadaver bone 7031, and 5.6 mm for cadaver bone 7168. The higher errors for individual measurements and higher standard deviation for grouped measurements are associated with larger soft tissue thicknesses, which is what we expect.

## 4. Discussion

This study investigated the detection limit of an improved portable XRF system for* in vivo* bone Pb quantification and validated the system using phantoms, goat bones, and human cadaver bones. The improved system geometry and detector size greatly enhanced the detection limit of the device and the ability of the device to accurately determine the concentration of Pb in bone especially at the* in vivo* situation.

The detection limit for the portable XRF device is improved from the previous portable XRF device by a factor of about 2. Through soft tissue thickness of 4 mm the device has the capability of a detection limit of 8 ppm, which is comparable to detection limit of 6–10 ppm with KXRF bone Pb measurement systems in most labs. It is also relevant to point out that this was with a 3-minute measurement time and that time could be increased by a factor of 2 or 3 to lower the detection limit further, while maintaining a reasonable radiation exposure. The main disadvantage of LXRF systems is the lack of penetration of the low energy X-rays and thus at depth, the ability of the system to determine concentration becomes limited. With this system, it is shown that even at depth of 4 mm the portable XRF device now has the capability of obtaining measurements in 3 minutes, which would be equivalent to a KXRF device with a 30-minute measurement. In studies it has been shown that tibia measurement sites with tissue thickness of less than 4 mm can be found on most seniors and about half of the general population [15, 22]. One of our target populations for this device is the senior people whose mobility might be confined by their health conditions. Another point of clarification is the fact that, with the low penetration depth of LXRF, the KXRF and portable XRF systems are sampling different sites of the bone. KXRF would be sampling the whole bone and LXRF would be sampling the superficial 0.5–1 mm of the bone. It is not very clear how Pb distributes over the layer of tibia bone and the literature on this topic is limited. Todd et al. showed higher concentrations of lead at 1-2 mm to the surface in bone [23], while Bellis et al. demonstrated a higher concentration of lead at a much thinner layer [24]. While the bone Pb concentrations from KXRF and LXRF are highly correlated in our study, further investigation with larger amount of samples is needed on the comparison for the absolute bone Pb concentrations from these two methods. It is also relevant to point out that with measurements of bone lead the goal is a correlation with health effect, which should be reflected in both surface and depth bone measurement sites.

The Pb concentrations found through KXRF and portable XRF measurements of bare bone show good correlation. In order for our device to determine the* in vivo* Pb concentration, our analysis methods should prove to be accurate with bone. Bone has different density and effective atomic number compared to our calibration phantoms, which led to differences in its resultant XRF spectrum. Our results with cadaver and goat bones show that our calibration methods adequately address these differences as the results are well correlated with KXRF data. In comparison, the traditional peak fitting calibration method results are shown with goat bones, and at larger Lucite thicknesses this method is worse than the background subtraction method. It is relevant to point out that other studies exploring the validity of LXRF for Pb studies used traditional peak fitting methods and showed that the results were not reliable especially at higher soft tissue thicknesses [21].

The background subtraction calibration method performed the best in our study. The bone adjustment method did correct the Compton peak to phantom values for a more equal comparison, but it fails to take into account the balance between the Compton peak, background, and signal, and because of this, at higher Lucite thicknesses, it exaggerates the problems seen with background subtraction. Bone calibration should be the best calibration method in theory, but due to the lack of standard bones with higher Pb concentrations, the calibration line for this method tended to produce results that were less accurate than the background subtraction method.

The correlation of bone Pb concentrations between KXRF and portable XRF is very good for phantoms and goat bones with Lucite thickness up to 5 mm, while the *R*-squared degraded a little for cadaver bones. This is mainly due to the small variation of the Pb concentration for these cadaver bones, as well as the difficulty to adjust the geometry of the bare bones. In measuring cadaver bones, the geometry presented issues if not strictly monitored. We found that the cadaver bones were prone to air gaps in the geometry, which led to significant changes in the spectrum caused by the increased distance without significant attenuation. Although this effect was visible in the bare bone data, given the geometry of* in vivo* measurements this effect would not be present as the bone is covered in soft tissue, so there will not be air gaps between the soft tissue and bone* in vivo*. Attenuation by soft tissue is accounted for with our calibration by determining the soft tissue thickness from the Compton peak, which in turn corrects for distance, as the gap between the detector and bone is filled with soft tissue.

Although only three intact cadaver bones were used to test the reproducibility of the system for bone Pb quantification and to validate the system in a real* in vivo* situation, several conclusions can be drawn from these limited data. First, this set of data confirmed the validity of the system for* in vivo* measurements, especially for the measurements with soft tissue thicknesses less than 5 mm. Second, the data confirmed that the thicknesses of the soft tissue significantly affect the uncertainties of the resultant concentrations. The standard deviations from the repeat measurements are lower than the uncertainties for individual measurements, which indicate that the uncertainties for individual measurements may be overestimated. In addition, the detection limit of the measurements calculated from the Pb concentration uncertainties (DL = 2 × sigma) for cadaver bones listed in Table 6 would be higher than those listed in Table 1 for corresponding soft tissue thicknesses. This is because the uncertainty calculated in Table 6 includes the error on the gross count and net count of the signal under the Pb L X-ray peak, while the DL calculated in Table 2 only includes error associated with the background of a blank phantom covered with the corresponding thicknesses of Lucite. Nonetheless, this data set shows an excellent agreement of bone Pb concentrations for cadaver bones at thickness of 1.3 and 4.1 mm, while the agreement deteriorates at 5.6 mm.

In the cadaver bone measurements, there is one bone (cadaver bone 6918), which we considered an outlier in our dataset. This bone came from a 100-year-old female and presents further challenges with the LXRF device. The spectrum from the bone had a much higher than normal Compton peak, which we attributed to the bone appearing more like soft tissue with respect to the spectral features. This resulted in a more pronounced overestimation of the background and worse signal quantification. In general the background subtraction method should overcome slight variations in bone between individuals, as the Compton peak will also relate back to the material of the bone. The relationship we had derived between the Compton peak and the Pb signal broke down for this particular bone. We have not isolated the characteristic that causes this issue but plan to look into bone density effects on the LXRF spectrum through simulation as well as our cadaver bone samples. Although bone 6918 has a Compton peak that is significantly different, other spectral features show differences that may be able to be exploited to correct the issues with the Compton peak in this spectrum.

The portable XRF system, now with a significantly lower detection limit, has its main advantages over KXRF with its portability, acquisition times, and ease of use. The new system can achieve a minimum detection limit equivalent to a KXRF measurement even through tissue thicknesses up to 5 mm. The portable XRF system also has the advantage of using an X-ray tube, which can be turned off when it is not in use and is less complicated for radiation license than a radioisotope source. The portable device lends itself for use in epidemiologic studies because of its quick measurement times and portability. The device allows for on-site Pb surveys and risk assessments of the environment, while performing exposure assessment of the community members.

In the future, the device can be improved by perfecting the data analysis algorithms for Pb as well as other metals. Monte Carlo methods could be used to accurately model the device and the spectrum of* in vivo* situations. This would help decrease the variability of measurements over different bone densities while also accounting for the tissue thickness over the bone. A main goal for the future of portable XRF technology would be applying it for the detection of other metals* in vivo*. The device can be used in collaboration with metal epidemiologists and toxicologists to study exposures and health effects of metals.

## 5. Conclusion

We have validated an advanced portable XRF system for* in vivo* bone Pb measurement and demonstrated the validity of using such a system to accurately quantify Pb in bone with soft tissue thickness up to 4-5 mm. The detection limit of the device with 4 mm of soft tissue is approximately the same as the detection limit of KXRF systems, and the novel analysis methods provide a better correlation for Pb quantification in bone samples. This device now has vast applicability in Pb exposure assessment in clinical and research settings.

## Figures and Tables

**Table 1:** Detection limit for bone Pb measurement by portable XRF devices at different soft tissue thicknesses.

Soft tissue thickness (mm)	Detection limit XL3t GOLDD+ (ppm)	Detection limit XL3 (ppm)
0 mm	1.2	2.0
1 mm	1.8	3.5
2 mm	2.9	5.9
3 mm	4.6	9.6
4 mm	8.0	12.8
5 mm	11.0	14.7

**Table 2:** Phantom Pb concentrations calculated using the background subtraction method.

Standard phantom ppm	Lucite thickness			
	0 mm	1 mm	2 mm	3 mm
0	−0.38 ± 0.8	−0.1 ± 1.3	−2.22 ± 2.1	3.31 ± 3.31
5	4.83 ± 0.86	3.57 ± 1.34	9.61 ± 2.19	7.37 ± 3.37
10	10.23 ± 0.9	10.5 ± 1.39	11.23 ± 2.19	17.83 ± 3.42
15	14.9 ± 0.94	13.3 ± 1.4	20.37 ± 2.27	13.39 ± 3.41
20	19.52 ± 0.97	19.17 ± 1.46	21.25 ± 2.27	17.37 ± 3.46
30	31.32 ± 1.06	30.09 ± 1.53	30.35 ± 2.36	32.34 ± 3.56
50	47.45 ± 1.17	52.76 ± 1.7	49.43 ± 2.48	48.54 ± 3.7
75	75.56 ± 1.33	77.44 ± 1.85	74.49 ± 2.65	74.34 ± 3.82
100	96.96 ± 1.44	108.65 ± 2.02	105.26 ± 2.84	101.73 ± 4.06

**Table 3:** Bone Pb concentrations for bare cadaver bone calculated using different calibration methods.

Cadaver bone ID	KXRF	Background subtraction	Bone calibration	Bone adjustment
6900	23.12	23.44	23.63	22.21
7202	22.17	19.23	19.94	18.48
6918	21.17	9.35	11.30	9.50
7131	20.77	25.59	25.53	24.34
7031	19.70	24.09	26.18	23.39
7162	18.36	17.91	18.81	17.48
7042	14.27	16.05	18.33	16.10
7002	13.54	15.29	16.48	15.15
6895	9.82	15.91	17.04	15.59
7168	3.64	1.08	3.82	−1.86

**Table 4:** Bone Pb concentrations for cadaver bone covered with 3 mm Lucite.

Cadaver bone ID	KXRF	Background subtraction	Bone calibration	Bone adjustment
6900	23.12	20.92	22.44	11.66
7202	22.17	16.72	18.53	8.14
6918	21.17	2.97	−1.35	−14.21
7131	20.77	18.10	11.76	9.61
7162	18.36	12.07	6.55	4.33
7002	13.54	10.80	13.55	3.25
6895	9.82	12.94	14.69	5.26

**Table 5:** Bone Pb concentrations for cadaver bones with different Lucite thicknesses calculated from the background subtraction method.

Cadaver bone	Background subtraction with Lucite thickness				
	KXRF	0 mm	1 mm	2 mm	3 mm
6900	23.12	23.44	24.54	19.49	20.92
7202	22.17	19.23	22.02	14.56	16.72
6918	21.17	9.35	13.05	5.96	2.97
7131	20.77	25.59	20.9	21.68	18.1
7162	18.36	17.91	18.5	16.32	12.07
7002	13.54	15.29	14.48	14.54	10.8
6895	9.82	15.91	13.86	10.31	12.94

**Table 6:** Bone Pb concentrations for three intact cadaver bones measured by portable XRF for 9 times compared to those measured by KXRF.

Cadaver bone ID	Cadaver bone (ppm)		
	7042	7031	7168
KXRF	14.27 ± 1.19	19.7 ± 1.04	3.64 ± 1.07

1	14.55 ± 1.68	17.69 ± 4.74	7.07 ± 10.51
2	15.42 ± 1.69	16.4 ± 4.72	5 ± 10.44
3	14.75 ± 1.68	17.75 ± 4.66	15.8 ± 10.67
4	12.58 ± 1.66	13.63 ± 4.68	12.93 ± 10.64
5	10.98 ± 1.65	22.86 ± 4.79	12.24 ± 10.57
6	14.69 ± 1.67	18.13 ± 4.87	16.2 ± 10.71
7	13.2 ± 1.66	24.15 ± 4.95	6.89 ± 10.54
8	12.44 ± 1.64	17.67 ± 5	11.58 ± 10.75
9	12.5 ± 1.64	15.55 ± 5.01	24.25 ± 10.76

Average ± SD	13.46 ± 1.46	18.2 ± 3.34	12.44 ± 5.93

Soft tissue thickness (mm)	1.3	4.1	5.6
